# Comparing metabolic syndrome parameters, oxidative stress, cellular metabolism, and gene polymorphism between people with schizophrenia and healthy controls

**DOI:** 10.3389/fpsyt.2026.1715991

**Published:** 2026-02-04

**Authors:** Khamelia Malik, Kresna Septiandy Runtuk, Nurmiati Amir, Martina Wiwie Nasrun, Mohammad Sadikin, Novi Silvia Hardiany, Abdul Halim Sadikin, Sondang P. Sirait

**Affiliations:** 1 Department of Psychiatry, Faculty of Medicine, Universitas Indonesia, Cipto Mangunkusumo, Jakarta, Indonesia; 2 Department of Psychiatry, Faculty of Medicine, Universitas Brawijaya, Universitas Brawijaya Hospital, Malang, Indonesia; 3 Department of Biochemistry and Molecular Biology, Faculty of Medicine, Universitas Indonesia, Jakarta, Indonesia; 4 Department of Dermatovenereology, Faculty of Medicine, Universitas Indonesia, Jakarta, Indonesia

**Keywords:** gene polymorphism, metabolic parameter, oxidative stress, PBMC, schizophrenia

## Abstract

**Background:**

Patients with schizophrenia have an increased risk of metabolic syndrome and cardiovascular disease, even in the absence of antipsychotic treatment. Oxidative stress, mitochondrial dysfunction, and genetic polymorphisms of the *GCLC* gene may contribute to this vulnerability.

**Methods:**

We conducted an analytical observational case–control study including 25 patients with schizophrenia (drug-naïve or drug-free) and 25 age- and sex-matched healthy controls, both groups predominantly men, recruited from two Indonesian psychiatric hospitals (2021–2023). Anthropometric (waist circumference, BMI, and blood pressure), metabolic (LDL-c, HDL-c, triglyceride, and HbA1c), and oxidative stress parameters (malondialdehyde [MDA], reduced glutathione [GSH], oxidized glutathione [GSSG], manganese superoxide dismutase [MnSOD], and adenosine triphosphate [ATP]) were measured from the blood sample. *GCLC* GAG trinucleotide repeat polymorphisms were analyzed by PCR.

**Results:**

Individuals with schizophrenia had significantly lower GSH and GSSG levels than controls but a higher GSH/GSSG ratio. Plasma MDA levels were lower in schizophrenia. High-risk GCLC genotypes were associated with higher MDA levels in schizophrenia. In the schizophrenia group, GSSG showed a moderate negative correlation with LDL-c (r = −0.430, p = 0.032). In the control group, MDA correlated positively with diastolic blood pressure (r = 0.411, p = 0.041) and GSSG correlated positively with triglycerides (r = 0.495, p = 0.012). No significant differences in ATP levels or mitochondrial activity were observed.

**Conclusion:**

Schizophrenia is associated with disruption of the glutathione system and early metabolic risk, influenced in part by *GCLC* GAG polymorphisms. These findings support the importance of early metabolic screening in schizophrenia.

## Introduction

Schizophrenia is a severe and complex psychiatric disorder characterized by disturbances in cognition, perception, emotional regulation, and behavior. It typically emerges in late adolescence or early adulthood and is associated with poor remission rates and reduced life expectancy (1, 2). In Indonesia, epidemiological data on schizophrenia remain limited, with a national prevalence of 1.8 per 1,000 population, higher in rural than urban areas (3). Twin studies have estimated the heritability of schizophrenia to be ~80%, underscoring the strong genetic contribution to its etiology (4).

Patients with schizophrenia have a two- to threefold increased mortality risk compared with the general population, with cardiovascular disease and cancer being the leading natural causes of death. A meta-analysis indicates that approximately one-third of patients with severe mental illness, including schizophrenia, are at increased risk for metabolic syndrome (MetS), even in the absence of antipsychotic treatment (5). Second-generation antipsychotics (SGAs) such as olanzapine, quetiapine, and risperidone are strongly associated with MetS (6).

Several local studies in Indonesia have reported a high prevalence of central obesity and other metabolic abnormalities among patients with schizophrenia, with central obesity affecting up to 74.5% of patients based on IDF criteria. Waist circumference and random blood glucose have been suggested as potential screening markers for MetS in this population (7, 8).

Oxidative stress, defined as an imbalance between reactive oxygen species (ROS) and antioxidant defenses, plays a critical role in the pathophysiology of many neuropsychiatric disorders, including schizophrenia and MetS (9). The brain’s high lipid content and relatively low antioxidant capacity make it particularly vulnerable to oxidative damage. Postmortem and serum studies have shown increased markers of oxidative stress, such as MDA and SOD, alongside decreased glutathione (GSH) levels in schizophrenia (9–15).

Cellular metabolism, particularly the production of ATP through mitochondrial oxidative phosphorylation, is essential for brain development, neuronal plasticity, and synaptic connectivity that underlie complex functions such as learning, memory, and cognition. In schizophrenia, alterations in ATP, phosphocreatine, and glucose metabolism—as well as reduced glycolytic enzyme activity—result in impaired bioenergetic processes that disrupt brain function (16).

Genetic variations, such as trinucleotide repeat (TNR) polymorphisms in the GCLC gene—encoding the catalytic subunit of glutamate-cysteine ligase, a key enzyme in GSH synthesis—may further increase susceptibility to oxidative stress in schizophrenia. Specific TNR GAG genotypes (e.g., 8/8, 8/9, 9/9) have been associated with redox dysregulation (17).

Peripheral blood mononuclear cells (PBMCs) have emerged as a practical and minimally invasive peripheral model for investigating oxidative stress and redox dysregulation in psychiatric disorders. PBMCs share several metabolic and antioxidant pathways with central nervous system tissues, including glutathione-related processes and mitochondrial redox mechanisms, making them a feasible surrogate for evaluating systemic oxidative imbalance in schizophrenia (18).

Despite emerging evidence, the pathophysiological links between schizophrenia, metabolic syndrome, oxidative stress, and cellular metabolism remain underexplored, particularly in low- and middle-income country contexts. Further investigation is essential to elucidate these mechanisms and identify potential biomarkers or therapeutic targets.

We hypothesized that the emergence of metabolic syndrome in individuals with schizophrenia occurs even before the initiation of antipsychotic treatment, potentially due to oxidative stress abnormalities, which may in turn be influenced by polymorphisms in the glutathione-regulating gene *GCLC*. Therefore, this study aimed to examine the relationships among these variables by comparing metabolic syndrome parameters between drug-naïve or drug-free individuals with schizophrenia and individuals without schizophrenia, in relation to oxidative stress (6, 19).

## Methods

### Study design

This research employed an analytical observational design to investigate biological and clinical variables associated with schizophrenia. The study was conducted from February 2021 to July 2023 at two sites: the Adult Psychiatry Clinic of Dr. Cipto Mangunkusumo National General Hospital (RSCM) and Dr. Soeharto Heerdjan Psychiatric Hospital, whereas laboratory samples were analyzed at Biochemistry Laboratory of the Faculty of Medicine, Universitas Indonesia. Ethical approval was granted by the Ethics Committee of the Faculty of Medicine, Universitas Indonesia (Ref: KET-1091/UN2.F1/ETIK/PPM.00.02/2020).

### Study sample

The target population consisted of patients with a confirmed diagnosis of schizophrenia. The accessible population included schizophrenia inpatients admitted to the acute psychiatric ward who demonstrated normal metabolic status (normal waist circumference, normal BMI, and normotension) and were either drug-naïve or drug-free, the latter defined as cessation of oral antipsychotics for at least 8 weeks or long-acting injectable antipsychotics for at least 12 weeks. Individuals without psychiatric diagnoses, confirmed using the Structured Clinical Interview for DSM-IV (SCID-I), served as controls. A total of 50 participants were recruited through non-probability consecutive sampling. The minimum required sample size was determined using the formula for comparing two independent means (unpaired t-test):


$$n=\frac{2{\left(Z_{\alpha /2}+Z_{\beta }\right)}^{2}\sigma^{2}}{{\text{Δ}}^{2}}$$

where $Z_{\alpha /2}=1.96$ for a 95% confidence level, $Z_{\beta }=0.84$ for 80% statistical power, σ represents the pooled standard deviation, and Δ is the expected difference in mean oxidative-stress parameters between groups. Based on pilot data, the pooled standard deviation was conservatively reduced and the expected mean difference increased, yielding a feasible minimum sample size of 25 participants per group. Although this requirement was met, recruitment was substantially limited by COVID-19 pandemic conditions. The study was conducted in a psychiatric hospital where most inpatients during this period were recurrent relapses, making the recruitment of drug-naïve or drug-free individuals—who were essential for controlling medication effects—particularly challenging. Despite these constraints, the calculated minimum sample size was successfully achieved.

### Study procedure

All participants who met the inclusion criteria were first introduced to the study’s objectives and procedures. After providing informed consent, obtained from participants and guardians, clinical data and biological samples were collected. Each participant underwent assessments that included the measurement of anthropometric parameters (body mass index and waist circumference), HbA1C, lipid profile, and blood pressure. Demographic data, including age, sex, marital status, income, education, family history of severe mental illness, family history of cardiometabolic disease, history of substance use, GCLC GAG TNR polymorphism, case criteria, subtype, and duration of symptoms, were also recorded. Clinical severity was assessed using the Positive and Negative Syndrome Scale (PANSS), and PANSS total scores are presented in the demographic table.

Metabolic syndrome (MetS) was assessed using the National Cholesterol Education Program Adult Treatment Panel III (NCEP ATP III) and the International Diabetes Federation (IDF) criteria. A diagnosis of MetS was made when participants met at least three of the following components:

1. Central obesity.
  ○ ATP III: waist circumference ≥102 cm (men) or ≥88 cm (women)
  ○ IDF: waist circumference ≥90 cm (men) or ≥80 cm (women) for Asian populations
2. Hypertriglyceridemia.
  ○ Triglycerides ≥150 mg/dL, or on specific treatment
  ○ Low HDL cholesterol<40 mg/dL in men or<50 mg/dL in women, or on treatment
3. Elevated blood pressure.
  ○ Systolic ≥130 mmHg, diastolic ≥85 mmHg, or on antihypertensive therapy
4. Impaired fasting glucose or abnormal glycemic marker.
  ○ ATP III/IDF: fasting plasma glucose ≥100 mg/dL
  ○ Additional parameter used in this study: HbA1c ≥5.7% indicating impaired glucose regulation

In addition to standard criteria, body mass index (BMI) was included as a supplementary metabolic indicator, with BMI ≥25 kg/m² considered overweight according to WHO Asia-Pacific cutoffs. Although BMI is not a core ATP III/IDF component, it was included to provide a more comprehensive assessment of metabolic risk in this population.

#### Specimen collection

Peripheral blood samples (10 mL) were collected approximately at 7–9 am before breakfast, using heparinized vacutainers for oxidative stress marker and non-heparinized vacutainers for metabolic parameters, after the participants had refrained from strenuous physical activity for at least 1 day. Both samples were collected from single blood collection. These samples were processed immediately: blood was centrifuged at 3,000g for 5 min at 4°C. The pellet was washed twice with 0.9% NaCl and stored at −80°C for PBMC analysis, whereas the plasma supernatant was aliquoted and frozen for MDA analysis.

#### PBMC isolation

PBMCs were used as the primary biological material for oxidative stress and metabolic analyses. Peripheral blood mononuclear cells (PBMCs) were isolated from whole blood by density gradient centrifugation using Ficoll. Blood diluted with PBS (1:1) was layered onto Ficoll and centrifuged at 400g for 30 min at room temperature. The mononuclear cell layer (buffy coat) was extracted, washed with PBS, and stored at −80°C for downstream analysis of oxidative stress and intracellular metabolism.

#### GCLC GAG TNR genotyping

Genotyping of the GCLC gene trinucleotide repeat (TNR) polymorphism was performed using PCR with specific primers: 5'-TTCTGCGGGCGGCTGAGTGTCC-3' (GAG strand) and 5'-ATGGCGCTTGGTTTCCTCCC-3' (CTC Strand) (17). DNA was extracted using Promega reagents, and PCR amplification was carried out with Promega GoTaq Master Mix. The thermal cycling protocol included an initial denaturation, followed by repeated cycles of denaturation, annealing, and extension, with a final extension step. The PCR products were separated on a 10% polyacrylamide gel, electrophoresed for 130 min at 100 V, and visualized using SilverXpress silver staining.

High-risk genotypes were defined as carriers of medium/long GAG repeat alleles (8/8, 8/9, 9/9), which have been associated with reduced GCLC activity and poorer glutathione synthesis. Low-risk genotypes were defined as individuals carrying at least one short allele (7/7 or 7/9), which is linked to preserved redox capacity.

#### Oxidative stress and metabolic marker measurement

Oxidative stress and metabolic markers were analyzed from the collected blood samples. Lipid peroxidation was measured using the Thiobarbituric Acid Reactive Substances (TBARS) assay based on the Uchiyama & Mihara method (20). Glutathione (GSH and GSSG) levels were determined colorimetrically using a commercial assay kit (Elabscience^®^, Cat. No. E-BC-K097-M). GSH was measured by mixing the sample (PBMC) with a protein precipitator, whereas GSSG was measured by mixing the cell sample with GSH scavenger solution, both with absorbance measured at 412 nm. Manganese superoxide dismutase (MnSOD) activity was evaluated using a xanthine oxidase inhibition method kit (RANSOD, Rx Monza, Cat. No. SD 125), with Cu/ZnSOD inhibited using sodium cyanide. The resulting MnSOD activity was expressed as percent inhibition and U/mg protein, read at 505 nm. Cellular ATP levels were quantified using a colorimetric ATP assay (Elabscience^®^, Cat. No. E-BC-K157-S), with measurements taken at 636 nm. All readings were conducted using a *Thermo Scientific Varioskan^®^ Flash* microplate reader, and assays were performed in duplicate to ensure reliability. The GSH/GSSG ratio reported in this study was calculated using mathematical division of GSH by GSSG values.

HbA1c, LDL, LDH, and triglycerides were quantified using standard clinical chemistry protocols performed in the Clinical Pathology Laboratory of Cipto Mangunkusumo Hospital (RSCM), employing validated automated analyzers. HbA1c was measured using high-performance liquid chromatography (HPLC). LDL, LDH, and triglycerides were analyzed using enzymatic colorimetric assays according to laboratory standard operating procedures.

### Data management and analysis

Data were analyzed using IBM SPSS Statistics version 26. For the difference between GCLC GAG TNR polymorphisms and metabolic syndrome parameters, analysis between patients and controls was assessed using independent t-tests or Mann–Whitney U tests, depending on data distribution. Similarly, the relationship between GCLC GAG TNR polymorphisms and oxidative stress markers as well as cellular metabolic activity was analyzed using the same statistical tests. A p-value of<0.05 was considered statistically significant. The correlation between MetS and oxidation stress parameter was analyzed using Spearman. P-values are presented with FDR adjustment using the Benjamini–Hochberg method.

## Results

### Study participant characteristics

Sociodemographic and clinical characteristics were assessed in 25 individuals with schizophrenia and 25 healthy controls using structured questionnaires (**Table 1**). Both groups were comparable in terms of age, sex, marital status, family history of severe mental illness, family history of cardiometabolic disease, substance use history, and GCLC GAG TNR polymorphism distribution, with no statistically significant differences observed across these variables (*p* > 0.05). Educational level and income differed significantly between patients and controls. These differences reflect the well-established social drift phenomenon, in which the chronic course of schizophrenia and associated cognitive decline reduce educational attainment and socioeconomic functioning over time.

**Table 1:** Demographics characteristic.

Parameter	Schizophrenia (n = 25)	Control (n = 25)	p-value	FDR p-value
**Age (years)**	31.84 ± 9.647	29.64 ± 8.859	0.302*	0.342
**Sex**	Male (n = 24) Female (n = 1)	Male (n = 22) Female (n = 3)	0.292^†^	0.342
**Marital status**	Unmarried, divorced (n = 20) Married (n = 5)	Unmarried, divorced (n=16) Married (n = 9)	0.208^†^	0.272
**Income**	Low income (n = 17) Lower-middle (n = 7) Upper-middle (n = 1) High (n = 0)	Low income (n = 1) Lower-middle (n = 22) Upper-middle (n = 1) High (n = 1)	**≤ 0.001^‡^**	**0.0012**
**Education**	High (n = 0) Middle (n = 11) Basic/Low (n = 14)	High (n = 10) Middle (n = 13) Basic/Low (n = 2)	**≤ 0.001^‡^**	**0.0012**
**Family history of severe mental illness**	Yes (n = 4) No (n = 21)	Yes (n = 1) No (n = 24)	0.157^†^	0.222
**Family history of cardiometabolic disease**	Yes (n = 6) No (n = 19)	Yes (n = 12) No (n = 13)	0.077^†^	0.119
**History of substance use**	Yes (n = 15) No (n = 10)	Yes (n = 16) No (n = 9)	0.771^†^	0.819
**GCLC GAG TNR polymorphism**	Low-risk (n = 9) High-risk (n = 16)	Low-risk (n = 9) High-risk (n = 16)	1.000^†^	1.000
PANSS (mean)				
Positive Negative General Total	20.84 ± 6.878 18.72 ± 10.953 37.04 ± 10.842 76.60 ± 17.416	7 7 16 30	**≤ 0.001** ^§^ **≤ 0.001** ^§^ **≤ 0.001** ^§^	**0.001** **0.001** **0.001**
**Case criteria**	*Drug naïve* (n = 9) *Drug free* (n = 16)	–	–	
**Subtype**	Paranoid (n = 14) Disorganized (n = 11) Catatonic (n = 0) Other (n = 0)	–	–	
**Duration of symptoms (years)** ^||^	1 (range: 0.5–38)	–	–	

Bold values indicate statistically significant results (p < 0.05).

*Mann–Whitney, ^†^Chi-square, **^‡^**Kruskal–Wallis, ^§^Independent T-test;

^||^Duration of illness = time from first psychotic symptoms to the date of assessment; presented as median (IQR) due to non-normal distribution.

### Metabolic syndrome profile in schizophrenia

A comparison of metabolic parameters between the schizophrenia group and healthy controls revealed several significant differences (**Table 2**). Participants with schizophrenia had a significantly smaller waist circumference (71.52 ± 8.88 cm) compared with controls (78.72 ± 10.45 cm; p = 0.025) and a lower body mass index (BMI) (19.85 ± 3.50 kg/m² *vs*. 23.21 ± 4.04 kg/m²; p = 0.003). In terms of lipid profile, the schizophrenia group exhibited lower LDL cholesterol levels (105.96 ± 27.27 mg/dL) compared with controls (127.44 ± 32.81 mg/dL; p = 0.022) and lower HDL cholesterol levels (40.96 ± 11.25 mg/dL) compared with controls (48.84 ± 11.03 mg/dL; p = 0.010). Conversely, triglyceride levels were significantly higher in the schizophrenia group (132.12 ± 49.36 mg/dL) than in healthy controls (111.96 ± 62.62 mg/dL; p = 0.038). No significant differences were observed for HbA1C, systolic blood pressure, or diastolic blood pressure between the two groups.

**Table 2:** Differences in metabolic parameters between schizophrenia and control groups.

Parameter/correlation	Schizophrenia (mean ± SD; n=25)	Control (mean ± SD; n=25)	*p*-value	FDR p-value
Between-group differences				
Waist circumference (cm)	71.52 ± 8.88	78.72 ± 10.45	**0.025**	0.075
BMI (kg/m²)	19.85 ± 3.50	23.21 ± 4.04	**0.003**	**0.024**
LDL-c (mg/dL)	105.96 ± 27.27	127.44 ± 32.81	**0.022**	0.075
HDL-c (mg/dL)	40.96 ± 11.25	48.84 ± 11.03	**0.010**	**0.04**
Triglycerides (mg/dL)	132.12 ± 49.36	111.96 ± 62.62	**0.038**	0.101
HbA1C	5.116 ± 0.433	5.120 ± 0.309	0.899	0.91
Systolic blood pressure	111.04 ± 13.065	111.76 ± 9.959	0.291	0.498
Diastolic blood pressure	75.84 ± 8.168	75.72 ± 5.631	0.499	0.665

Bold values indicate statistically significant results (p < 0.05).

Correlation analyses were performed to explore the relationship between oxidative stress markers and metabolic parameters. Within the schizophrenia group, oxidized glutathione (GSSG) demonstrated a significant moderate negative correlation with LDL cholesterol (r = –0.430, p = 0.032), indicating that higher LDL levels were associated with lower GSSG concentrations. In the control group, two significant associations were observed. First, malondialdehyde (MDA) levels were moderately and positively correlated with diastolic blood pressure (r = 0.411, p = 0.041), suggesting a link between lipid peroxidation and vascular function. Second, GSSG levels were moderately and positively correlated with triglyceride levels (r = 0.495, p = 0.012), indicating a potential relationship between oxidative glutathione imbalance and lipid metabolism. No other oxidative stress or cellular metabolism markers showed statistically significant correlations with metabolic syndrome parameters in these groups. Genotype-based analysis also show no significant differences in both group.

### Oxidative stress activity and cellular metabolism in schizophrenia patients and its association with GCLC GAG TNR polymorphism

Oxidative stress markers were evaluated in individuals with schizophrenia and healthy controls (**Table 3**). A significant difference was observed in MDA levels between groups: Individuals with schizophrenia had lower MDA levels (0.690 ± 0.471 nmol/mL) compared with controls (0.818 ± 0.242 nmol/mL; *p* = 0.013). Subgroup analysis based on GCLC GAG TNR genotypes revealed that schizophrenia patients with high-risk genotypes (8/8, 8/9, 9/9) had higher MDA levels than those with low-risk genotypes (7/7, 7/9; 0.476 ± 0.197 nmol/mL), although the difference approached but did not reach statistical significance (*p* = 0.054).

**Table 3:** Comparison of oxidative stress parameter and ATP between schizophrenia and controls.

Marker	Schizophrenia (mean ± SD; n=25)	Controls (mean ± SD; n=25)	*p*-value	FDR p-value
MDA (nmol/mL)	0.690 ± 0.471	0.818 ± 0.242	**0.006**	**0.036**
GSH (µmol/10^6^)	446.09 ± 354.79	1325.49 ± 482.54	**<0.001**	**0.0012**
GSSG (µmol/10^6^)	101.622 ± 116.89	381.26 ± 169.30	**<0.001**	**0.0012**
GSH/GSSG Ratio	17.036 ± 48.02	5.832 ± 6.917	0.171	0.315
MnSOD (U/mg)	0.997 ± 0.809	0.829 ± 0.305	0.600	0.757
ATP (mmol/10^9^)	736.86 ± 969.79	508.68 ± 394.33	0.771	0.91

Bold values indicate statistically significant results (p < 0.05).

Levels of reduced glutathione (GSH) were significantly lower in the schizophrenia group (446.09 ± 354.79 µmol/10^6^) compared with controls (1325.49 ± 482.54 µmol/10^6^; *p* < 0.001). Oxidized glutathione (GSSG) was also lower in the schizophrenia group (101.622 ± 116.89 µmol/10^6^) compared with controls (381.26 ± 169.30 µmol/10^6^; *p* < 0.001). GSH/GSSG ratio was markedly higher in schizophrenia (17.036 ± 48.02) than in controls (5.832 ± 6.917), but show no significant differences.

Subgroup analysis within the schizophrenia group showed no significant difference in GSH levels by genotype (*p* = 0.384) (**Table 4**). However, individuals with high-risk genotypes had slightly lower GSH (391.05 ± 295.69 µmol/10^6^) than those with low-risk genotypes (543.95 ± 443.69 µmol/10^6^). However, GSSG levels were significantly lower in high-risk genotype carriers (68.92 ± 78.37 µmol/10^6^) compared with low-risk (159.76 ± 153.42 µmol/10^6^; *p* = 0.010). Although the GSH/GSSG ratio tended to be higher in high-risk individuals (24.12 ± 59.44) than in low-risk (4.45 ± 3.73), this difference did not reach statistical significance. No significant differences were found in MDA, MnSOD, GSH, GSSG, or the GSH/GSSG ratio between genotype groups in the control population. For mitochondrial antioxidant enzyme activity, cellular metabolism activity, as indicated by intracellular ATP concentration, did not differ significantly between individuals with schizophrenia and healthy controls. Subgroup analysis by GCLC GAG TNR genotype also revealed no significant differences in ATP levels within the schizophrenia group or the control group.

**Table 4:** Comparison of oxidative stress parameters and cellular metabolism between low-risk and high-risk genotypes in schizophrenia and control groups.

Marker	Low-risk (mean ± SD; n=25)	High-risk (mean ± SD; n=25)	*p*-value	FDR p-value
Schizophrenia				
MDA (nmol/mL)	0.476 ± 0.197	0.810 ± 0.540	0.054	0.129
GSH (µmol/10^6^)	543.95 ± 443.69	391.05 ± 295.69	0.384	0.614
GSSG (µmol/10^6^)	159.76 ± 153.42	68.92 ± 78.37	**0.010**	**0.04**
GSH/GSSG Ratio	4.45 ± 3.73	24.12 ± 59.44	0.084	0.183
MnSOD (U/mg)	0.955 ± 1.169	1.021 ± 0.562	0.308	0.614
ATP (mmol/10^9^)	452.22 ± 533.99	896.96 ± 1129.93	0.910	0.91
Control				
MDA (nmol/mL)	0.855 ± 0.148	0.798 ± 0.284	0.910	0.91
GSH (µmol/10^6^)	1,313.434 ± 526.594	1,332.272 ± 473.837	0.803	0.91
GSSG (µmol/10^6^)	434.033 ± 88.931	351.576 ± 197.564	0.452	0.638
GSH/GSSG Ratio	0.893 ± 0.283	0.793 ± 0.32	0.445	0.638
MnSOD(U/mg)	0.894 ± 0.284	0.794 ± 0.32	0.335	0.614
ATP (mmol/10^9^)	348.88 ± 380.81	598.57 ± 384.08	0.095	0.19

Bold values indicate statistically significant results (p < 0.05).

### Oxidative stress and cellular metabolism of *peripheral blood mononuclear cells*

**Figure 1:**
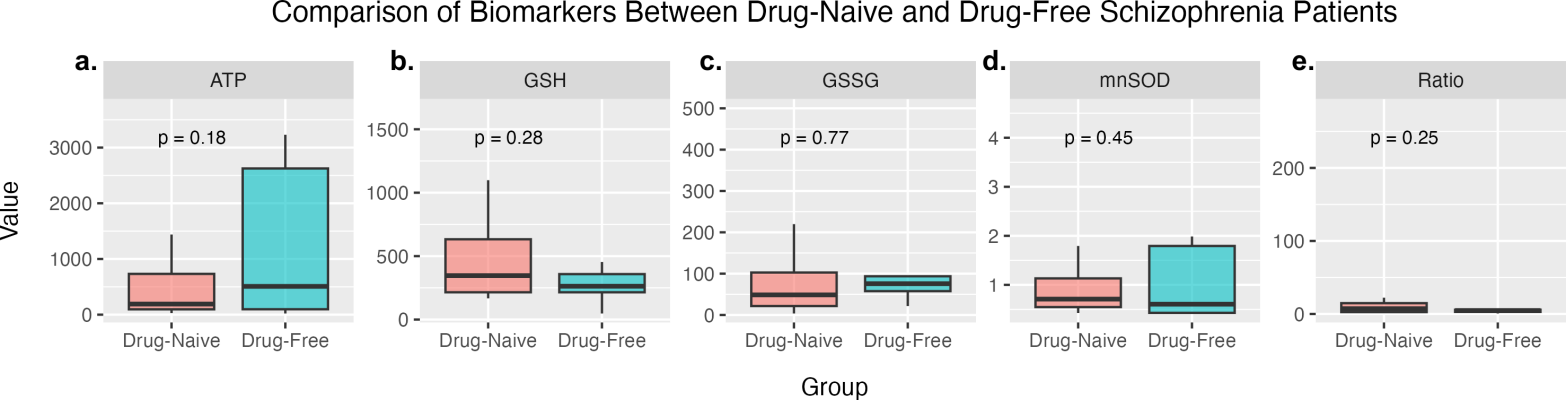
*Drug naïve* and *drug-free* comparison in PBMC cells of people with schizophrenia **(a)** ATP level, **(b)** GSH level, **(c)** GSSG level, **(d)** MnSOD level, (e) GSH/GSSG ratio.

We analyze the activity level of PBMC in *the drug-naïve and drug-free groups.* There is no difference in activity found in both groups (**Figure 1**) in MnSOD (*p=*0.257*)*, ATP *(p=*0.914*)*, GSH *(p=*0.610*)*, GSSG (*p* = 0.188), and GSH–GSSG ratio (*p=*0.109). There is no influence of a history of antipsychotic use between drug-free and drug-naïve populations.

## Discussion

This study examined oxidative stress, cellular metabolism, and the influence of GCLC GAG TNR polymorphisms in schizophrenia, using peripheral blood mononuclear cells (PBMCs). PBMCs are widely used in neuropsychiatric research as a minimally invasive model for assessing systemic oxidative stress. Although PBMCs do not perfectly represent neuronal metabolism, they reflect peripheral redox activity and have been consistently shown to align with oxidative abnormalities observed in schizophrenia.

Patients had significantly lower GSH and GSSG levels than controls, with a higher but not significant increase GSH/GSSG ratio. This finding consistent with a recent study that shows a decreased GSSG level and an elevated GSH/GSSG ratio in acute relapsed schizophrenia (21). The PBMC findings parallel reductions reported in central nervous system studies using proton magnetic resonance spectroscopy (¹H-MRS) and in peripheral tissue, supporting a generalized redox imbalance in schizophrenia (22). GCLC GAG TNR polymorphisms influenced GSSG levels in patients—low-risk genotypes had higher GSSG than high-risk carriers—suggesting a genotype-dependent effect on oxidative stress pathways. No genotype effects were seen for GSH or the GSH/GSSG ratio, and no associations were present in controls, indicating this genetic influence may be specific to schizophrenia. This finding contrasts with prior work showing that high-risk GCLC alleles are associated with impaired GSH synthesis and heightened oxidative susceptibility (17). Our results of reduced GSH and GSSG but altered ratios are consistent with meta-analyses and postmortem studies reporting redox imbalance in schizophrenia, including elevated MDA and altered antioxidant enzyme activities (9, 10, 22, 23).

Contrary to some reports of increased oxidative damage, patients had lower plasma MDA than controls. Differences in sample type, treatment status, or measurement methods may explain this. Within the schizophrenia group, high-risk GCLC genotypes were linked to higher MDA, suggesting greater vulnerability to oxidative membrane damage in this subgroup (24–26).

ATP-based assessment of cellular metabolism showed no significant differences between patients and controls, nor genotype effects, indicating preserved ATP production in PBMC. This contrasts with mitochondrial bioenergetic changes seen in other models, such as induced pluripotent stem cell-derived neurons, and with findings in brain tissue showing elevated ATP, phosphocreatine level changes, and glucose metabolism in prefrontal and temporal regions, which correlate with negative symptoms and neuropsychological deficits (16, 27–29). These disturbances reflect impaired oxidative phosphorylation with a shift toward anaerobic respiration, elevated lactate, reduced pH, and decreased activity of glycolytic enzymes such as hexokinase and phosphofructokinase in the dorsolateral prefrontal cortex (30). Together, these results suggest that while PBMC may maintain basal ATP production, neuronal bioenergetic deficits become evident under stress or in brain regions critical for cognition.

Metabolic syndrome (MetS) profiles revealed an unusual pattern: despite lower BMI, waist circumference, LDL-c, and HDL-c than controls, patients with schizophrenia had higher triglyceride levels. This supports the idea that metabolic risk in schizophrenia may be intrinsic to the illness and present early, rather than solely due to obesity or antipsychotic use. GCLC polymorphisms were not associated with any MetS parameters in either group. The higher triglycerides despite lower BMI parallels previous epidemiological studies showing metabolic risk as an intrinsic feature of schizophrenia, independent of antipsychotic use (5, 6, 31). Local studies similarly demonstrated central obesity and glucose dysregulation in Indonesian cohorts. However, our results did not report the same findings. This discrepancy could be attributable to differences in study design, as the previous study did not restrict its participants to drug-naïve or drug-free patients. Antipsychotic exposure is known to markedly increase BMI and central adiposity, whereas our strict metabolic inclusion criteria resulted in a leaner patient sample (7, 8). Systematic reviews and other studies confirm that schizophrenia patients have higher prevalence of metabolic syndrome even independent of medication use (15, 32).

Within people with schizophrenia, oxidative stress markers showed selective metabolic associations: MDA correlated moderately with diastolic blood pressure, and GSSG negatively correlated with LDL-c. These findings align with evidence linking oxidative stress to cardiovascular risk and may help explain the high cardiometabolic burden in schizophrenia (11). Other markers (MnSOD, GSH, GSH/GSSG ratio, and ATP) showed no significant metabolic correlations, suggesting pathway-specific rather than global oxidative-metabolic links.

We also analyze the difference between drug-naïve and drug-free people living with schizophrenia. We found no significant differences between drug-naïve and drug-free patients, supporting the idea that redox abnormalities are intrinsic to schizophrenia rather than medication-driven. However, a trend toward higher GSH in drug-free patients raises the possibility of long-term antipsychotic effects on antioxidant systems, consistent with preclinical studies showing certain antipsychotics enhance antioxidant defenses via BDNF and ERK/Akt pathway activation (33, 34). Conversely, some studies have shown that certain antipsychotics exacerbate oxidative stress parameters (35).

In summary, schizophrenia is characterized by systemic redox imbalance and distinctive metabolic alterations, partly modulated by GCLC GAG TNR polymorphisms. Associations between oxidative stress markers and cardiometabolic parameters highlight the importance of integrated metabolic and oxidative monitoring in clinical care. Longitudinal studies with cell-type-specific analyses and real-time metabolic flux measurements are warranted to clarify mechanisms and guide therapeutic strategies.

### Limitations

This study has several limitations that should be acknowledged. First, a wide range and high variability were observed in oxidative stress measures in PBMCs, including MDA, GSH, GSSG, MnSOD, and ATP. This variability likely reflects multifactorial influences, such as individual biological heterogeneity, differences in redox regulation across patients, and technical variability inherent to oxidative stress assays. Because these factors are difficult to fully control in clinical samples, the variability should be considered an important limitation of this study. Exploratory ANOVA models examining genotype×group interactions were considered; however, these analyses were not conducted because the control group showed no significant variability across oxidative markers, limiting the interpretability of interaction effects.

Second, among drug-free individuals, we did not perform detailed analyses of the types or duration of previous antipsychotic exposure. Different antipsychotic agents may have heterogeneous effects on oxidative stress and metabolic parameters, which could not be fully accounted for in this study.

Third, lifestyle factors such as tobacco use, alcohol consumption, and other substance exposures were not controlled. These variables may influence oxidative stress and metabolic outcomes but could not be standardized during the pandemic-restricted recruitment period.

Fourth, fasting plasma glucose—a standard diagnostic criterion for metabolic syndrome—was not obtained due to logistical constraints. Instead, HbA1c was used as an alternative glycemic marker, which reflects longer-term glucose regulation but may differ from fasting glucose in sensitivity to acute metabolic changes.

## Data Availability

The raw data supporting the conclusions of this article will be made available by the authors, without undue reservation.
